# Minireview: Nonsteroidal anti-inflammatory drugs in colorectal cancer: from prevention to therapy

**DOI:** 10.1038/sj.bjc.6600829

**Published:** 2003-03-18

**Authors:** P Ricchi, R Zarrilli, A di Palma, A M Acquaviva

**Affiliations:** 1 Dipartimento di Biologia e Patologia Cellulare e Molecolare ‘L. Califano’, Istituto di Endocrinologia ed Oncologia Sperimentale ‘G. Salvatore’ del Consiglio Nazionale delle Ricerche, Facoltà di Medicina e Chirurgia, Università ‘Federico II’, via S. Pansini 5, Napoli 80131, Italy

**Keywords:** NSAIDs, anticancer drugs, colon cancer, chemoprevention

## Abstract

In this review, we discuss the available experimental evidences supporting the chemopreventive efficacy of nonsteroidal anti-inflammatory drugs (NSAIDs) on colorectal cancer and the biological basis for their possible role as anticancer agents. Although the comprehension of the mechanisms underlying the effects of these drugs on colon cancer cells is incomplete, research efforts in identifying the biochemical pathway by which NSAIDs exert their chemopreventive effect have provided a rationale for the potential use of NSAIDs alone or in combination with conventional and experimental anticancer agents in the treatment of colorectal cancer. In this paper, we review three main issues: (i) the role of COX-2 in colon cancer; (ii) the common death pathways between NSAIDs and anticancer drugs; and (iii) the biological basis for the combination therapy with COX-2 selective inhibitors and new selective inhibitors of growth factor signal transduction pathways.

## ROLE OF COX-2 IN COLON CANCER

Colorectal cancer is the third most common cancer in the world, and the second most common cause of cancer-related death. Despite the development of new strategies of aggressive surgical and adjuvant therapy, little progress has been made in the successful management of advanced disease; therefore, much hope is currently placed on chemoprevention.

A large body of evidence from epidemiological studies and clinical trials in patients with the hereditary colon cancer syndrome, familial adenomatous polyposis coli (FAP) indicates that aspirin and related drugs, known as nonsteroidal anti-inflammatory drugs (NSAIDs), which share the property of inhibiting the cyclooxygenase (COX) enzyme, hinder the development of colon cancer and perhaps other cancers as well.

COX is the rate-limiting enzyme for the synthesis of eicosanoids, such as prostaglandins, from arachidonic acid. Two COX isoforms have been identified: the constitutively expressed COX-1 and the inducible COX-2. COX-1 is a housekeeping gene and has an important role in protecting the gastroduodenal mucosa. The COX-2 gene, an immediate-early response gene, is rapidly induced in response to tumour promoters, cytokines, and growth factors (DuBois *et al*, 1994; Di Popolo *et al*, 2000). NSAIDs may achieve different degrees of inhibition of COX-1 and COX-2 and can be grouped into selective and nonselective inhibitors of COX-2.

The development of COX-2-specific inhibitors, like celecoxib and rofecoxib, drugs that maintain their anti-inflammatory properties while preserving the biosynthesis of protective prostaglandins, further raised interest in this field. A large body of research was therefore performed to clarify the relative involvement of the two COX isoforms in colorectal carcinogenesis and the role of COX-2-selective inhibitors as chemopreventive agents.

COX-2, but not COX-1, expression was found to be increased in colorectal cancer (Eberhart *et al*, 1994): approximately 50% of adenomas and 80–85% of adenocarcinomas showed increased expression of COX-2. This observation was later confirmed by other investigators. More recently, studies underlying the role of COX-2 in colorectal carcinogenesis were performed in animal models: a series of reports documented that COX-2 mRNA was elevated both in chemically induced colon tumours and intestinal tumours in experimental animal models, and that traditional NSAIDs and new COX-2-selective inhibitors reduced the number of polyps and tumour incidence (Gupta and Dubois, 2001). Although these data indicate that COX-2 plays a wide role in colorectal carcinogenesis and that its pharmacological inhibition by NSAIDs is the central event in the chemoprevention of colon cancer, the mechanisms involved are yet to be clarified. It is also unclear how COX-2 elevation exerts its oncogenic effect at the molecular level, but an expanding body of evidence indicates a pivotal role of COX-2 elevation in the prevention of programmed cell death, that is, apoptosis, a process by which damaged or mutated cells in colonic mucosa are removed (Cao and Prescott, 2002).

## COMMON DEATH PATHWAYS BETWEEN NSAIDS AND ANTICANCER DRUGS

**Figure 1:**
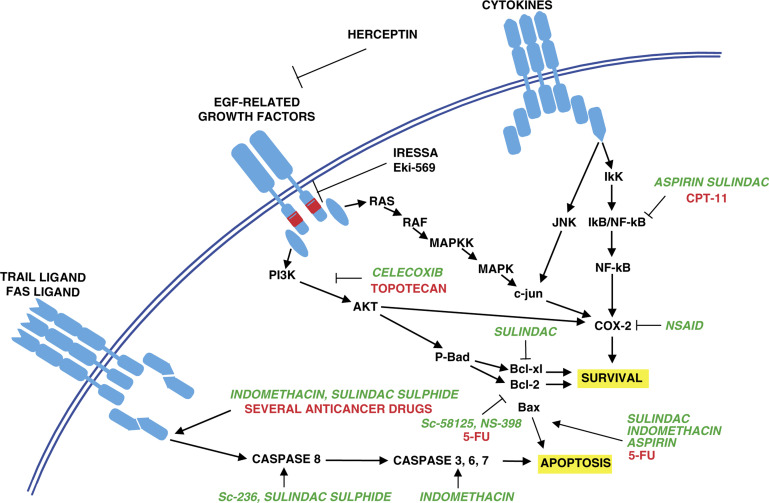
Molecular mechanisms that mediate the effects of NSAIDs and anticancer drugs on survival and apoptosis in colon cancer cells. Schematic representation of cytokine, EGF-related growth factors and TRAIL ligand-dependent signal transduction pathways for survival and apoptosis. Stimulatory and inhibitory effects are indicated by arrows and bars, respectively. Abbreviations: MAPK=mitogen-activated protein kinase, MAPKK=mitogen-activated protein kinase kinase, JNK=jun kinase, IkB=inhibitor kinase B, NF-kB=nuclear factor kappa B, COX=cyclooxygenase, PI3K=phosphatidylinositol 3 kinase.

Today, the management of advanced colorectal cancer involves mainly the use of chemotherapeutic drugs. For this purpose, 5-FU or new compounds direct thymidylate synthase (TS) inhibitors like raltitrexed and nolatrexed, or irinotecan (CPT-11) and oxaliplatin, have established activity both as single agents and in combination. Most of these current available cytotoxic drugs have well-recognised intracellular macromolecular targets (i.e. TS for 5-FU, toposisomerase 1 for CPT-11). However, the pattern and the extent of cell damage induced by chemotherapy in human cancer cells have been suggested to depend also on the pathway downstream from drug–target interaction that, once triggered, will initiate apoptosis (Canman *et al*, 1992). Apoptosis is a complicated process and its proper execution programme requires the coordinated activation and execution of several pathways. There is emerging evidence both that the major target for anticancer therapy is the induction of apoptotic cell death, and that resistance to antitumour treatments relies on reduced sensitivity to apoptosis induction. Hence, novel treatment approaches have tried to overcome this kind of drug resistance by manipulating key regulator proteins in apoptosis machinery (Nicholson, 2000). Figure 1

summarises the major apoptotic pathway in mammalian cells and the current recognised sites of interference of COX inhibitors.

In order to understand the molecular mechanisms underlying the chemopreventive effect of NSAIDs, a number of studies have assessed the effect of NSAIDs on cell growth and apoptosis in cultured colon cancer cells. We and others have shown that nonselective and COX-2-selective inhibitors are able to inhibit cell growth and induce apoptosis in colon cancer cells (Elder *et al*, 1997; Ricchi *et al*, 1997; Di Popolo *et al*, 2000).

This effect *in vitro* has been initially attributed to COX inhibition and to the consequent reduction in prostaglandin levels. On the other hand, an expanding literature shows a reduced role of the prostaglandin pathway in the mechanism of action of NSAIDs and new coxib (Song *et al*, 2002). In fact, several NSAIDs maintain their properties also in COX-2-negative tumour cell lines and independently from COX-2 expression levels (Elder *et al*, 1997) and, between coxib, Vioxx, although exhibiting similar COX-2 selectivity and clinical efficacy for inflammatory indication with respect to celecoxib, has little or no anticancer activity (Johnson *et al*, 2001; Waskewich *et al*, 2002). Although the concentration of NSAIDs required to show these effects *in vitro* is sometimes higher than the amount required to inhibit prostaglandin production and higher than that achievable *in vivo*, numerous studies have clearly focused on the induction of apoptosis as the mechanism for growth inhibition of colon cancer cells. Interestingly, most of the death pathways affected by NSAIDs are those involved in anticancer drug-induced apoptosis and resistance (Figure 1).

In fact, high doses of aspirin were shown to antagonise the survival signalling pathway controlled by the transcriptional factor NF-kB (Kopp and Ghosh, 1994) through the inhibition and the destruction of the activity of IKB kinase *β* (Yin *et al*, 1998), an enzyme that activates the NF-kB pathway by phosphorylating the inhibitory subunit of NF-kB (IkB). A similar effect on inhibition of IKB kinase *β* activity was reported for sulindac (Yamamoto *et al*, 1999). On the other hand, one study reported that inhibition of NF-kB through the adenoviral delivery of a modified form of IkBa sensitised chemoresistant tumours to the apoptotic effect of the antiblastic compound irinotecan (Wang *et al*, 1999).

Recently, it has been shown that celecoxib-induced apoptosis was mediated by the inhibition of the activation (phosphorylation) of the antiapoptotic protein kinase AKT (Hsu *et al*, 2000). A related study demonstrated that transfection of the constitutively active akt cDNA in a human lung cancer cell (A549) resulted in the reduction of the cytotoxic effect of topotecan, and concluded that topotecan exhibited its toxic effects by downregulating the Akt survival signalling pathway (Nakashio *et al*, 2000).

In eucaryotic cells, survival and apoptosis are often regulated by relative level of members of the Bcl-2 family proteins. One study reported that members of the Bcl-2 family of proteins in human colon cancer cell lines were modulated by 5-FU and that the ratio of the antiapoptotic protein bcl-xl to the proapoptotic protein bax may be related to chemosensitivity (Nita *et al*, 1998). On the other hand, the COX-2-selective inhibitors SC-58125 and NS-398 were reported to sensitise colon and prostate cancer cells, respectively, to apoptosis by downregulating the antiapoptotic protein bcl-2 (Liu *et al*, 1998). In addition, in a study performed in HCT 116 colorectal cancer cells, the proapoptotic protein bax had a pivotal role both in 5-FU and sulindac-induced apoptosis, and several NSAIDs could decrease the level of the antiapoptotic gene BCL-xl (Zhang *et al*, 2000). Similarly, aspirin and indomethacin were shown to induce apoptosis through upregulation of the proapoptotic proteins bax and bak and activation of caspase 3 (Zhou *et al*, 2001).

Several evidences suggest that both TRAIL (tumour necrosis factor-related apoptosis-inducing ligand) and FAS signalling pathways are involved in chemotherapy-induced apoptosis, either activating the initiator caspase 8 or at the level of downstream effector caspase (caspases 3, 6, 7) activation (Petak and Houghton, 2001). In the NSAID field, it was reported that indomethacin and sulindac sulphide induced apoptosis of human leukemic Jurkat cells by a mechanism that required the Fas-associated death domain (Han *et al*, 2001). Similarly, a recent paper demonstrated that sulindac sulphide and the COX-2-selective inhibitor SC-′236 could induce apoptosis coupled with upregulation of DR5, caspase 8 activation, and Bid cleavage in HCT116 human colon cancer cells (He *et al*, 2002). Thus, the common interference of anticancer agents and NSAIDs with the apoptosis machinery and signalling, independently from their COX inhibitory effect supports their use either as a single agent or as modulators of the cell death signalling pathway to potentiate or restore cytotoxic anticancer drug action.

The evidence of NSAID proapoptotic and growth inhibitory effects prompted the evaluation of the *in vitro* effects of a combination treatment with conventional anticancer drugs. Synergistic effects were reported as early as in the 1980s. The combination of indomethacin with methotrexate increased the killing of cultured NC cells without providing an exhaustive mechanism for this effect (Bennett *et al*, 1987). More recently, a report performed the first extensive screen of commercially available NSAIDs with anticancer drugs and discussed the potential clinical benefits of such a combination (Duffy *et al*, 1998); the authors observed dose-dependent synergistic effects among the two classes of drugs and concluded that this effect occurred independently from COX inhibition. Further combination therapies were evaluated: either sulindac sulphide, a COX-1 and COX-2 inhibitor, or other compounds devoid of COX-inhibitor property, in combination with paclitaxel and cisplatin, produced synergistic activity against three NSCLC and SCLS cell lines (Soriano *et al*, 1999). More recently, it was found that nimesulide, a COX-2-selective inhibitor, at clinically achievable concentration reduced the IC_50_ values of various anticancer agents in a lung cancer line (Hida *et al*, 2000).

Further studies are needed to better evaluate the combination therapy of cytotoxic drugs and new COX-2 inhibitors other than celecoxib and rofecoxib in colorectal cancer cells to induce the optimal apoptotic effect and to avoid reduction of anticancer drugs activity in the case of concurrent administration. In fact, several conventional NSAIDs and currently used COX-2 inhibitors are mainly antiproliferative and may upregulate the cyclin-dependent kinase inhibitors p27^Kip1^ and p21^WAF1^ (Hung *et al*, 2000; King and Khalili, 2001), thus theoretically limiting the effectiveness of cell cycle-dependent chemotherapeutic agents. In partial support of this hypothesis, we have described that high doses of aspirin and NS-398 were able to reduce CPT-11- and Vp-16-induced toxicity in cultured colon cancer cells independently of their COXs isoform profile expression (Ricchi *et al*, 2002).

A potential role for NSAIDs in an adjuvant setting can also be hypothesised independently from their effects on cell growth and survival. Several promising classes of noncytotoxic agent are under intense investigation (Sobrero *et al*, 2000). Most of them target the well-recognised series of steps involved in the process of metastasis. Neoangiogenesis is a crucial event of tumorigenesis and metastatic development, because neovascularisation is required for tumours to grow beyond 2–3 mm in size. There is mounting evidence that prostaglandins participate in angiogenesis, regulating the production of proangiogenic factors such as vascular endothelial growth factor (VEGF). Both COX-1 and COX-2 are involved in tumour vascularisation, and COX-2 inhibitors can directly affect angiogenesis (Gately, 2000). A recent observation suggested that angiogenesis was directly involved in the biology of tumour dormancy and was associated with the risk of recurrence after adjuvant therapy in breast cancer (Gasparini *et al*, 2001). It was also found that angiogenic inhibitor TNP inhibited tumour growth when administered following cyclophosphamide (Shusterman *et al*, 2001); the authors also documented that TNP directly inhibited angiogenesis and increased apoptotic index in treated tumours, particularly in the setting of minimal or subclinical disease. Finally, a recent paper demonstrated that NSAIDs were able to suppress PGE_2_- and PGI-mediated endothelial-cell spreading and migration *in vitro* and fibroblast growth factor-2-induced angiogenesis *in vivo* (Dormond *et al*, 2001).

Despite all the discussed evidences of NSAID interference at different levels with tumour cell proliferation, survival, and metastatic formation, only few reports are available in the literature concerning the treatment of human clinical cancer by NSAIDs. In 1994, Lundholm *et al*, in a randomised trial, treated 135 undernourished patients with metastatic solid tumours to receive placebo, prednisolone, or indomethacin, until death. Indomethacin-treated patients suffered less pain and had a significant increase in survival (Lundholm *et al*, 1994). Survival analysis from all patients treated with either indomethacin or prednisolone demonstrated a significantly prolonged survival by anti-inflammatory treatment compared with placebo treatment. They did not observe any serious relevant side effect for indomethacin treatment and propounded for indomethacin with respect to prednisolone, based on the theoretically and experimentally less catabolic and immunoattenuating effect of indomethacin on the host compared with glucocorticoids.

In 1996, Sinicrope *et al* conducted a phase I trial in 15 patients who had failed prior 5-FU-based therapy (Sinicrope *et al*, 1996). They administered sulindac (300 mg day^−1^) associated with the same schedules of systemic 5-FU and *per os* levamisole used in the intergroup adjuvant trial (450 mg m^−2^ 5-FU+150 mg daily levamisole). Leukopenia was no more frequent than in patients receiving 5-FU and levamisole in the intergroup adjuvant trial. All toxic effects were reversible, and there were no chemotherapy-related deaths. A partial response was seen in one patient, three patients had disease stabilisation, and 10 patients progressed while in the study. Uptill today, celecoxib has been successfully used in randomised clinical trials only as a chemopreventive agent to treat colorectal and duodenal polyps in FAP patients (Steinbach *et al*, 2000; Phillips *et al*, 2002); the two doses (100 and 400 mg twice daily) of celecoxib employed were well tolerated compared with placebo, but in the groups of patients receiving the most effective dose (400 mg twice daily), one allergic reaction and a greater incidence of adverse effects (dyspepsia and abdominal pain) were observed.

New trials are currently evaluating coxibs as single agents to treat only *other* precancerous lesions and cancer that express COX-2 in adjuvant settings (Thun *et al*, 2002). However, all data discussed above may support the use of NSAIDs and new coxibs for the treatment of cancer in combination with standard therapy also independently from cancer cell COX-2 levels.

Phase I studies are needed to evaluate *in vivo* the optimal dose and timing of conventional and new COX-2-selective NSAIDs in combination with the currently used chemotherapy regimen for subsequent use in phase II trials.

## FURTHER ATTRACTIVE COMBINATION THERAPY

The recent development of inhibitors of biochemical pathways that are altered in cancer cells has given rise to the possibility of evaluating new combination treatment regimens. In colon cancer cells, different growth factors such as EGF, IGFs have been identified as positive regulators of cell growth. In particular, the erb/HER pathway seems to play an important role in the maintenance of neoplastic disease. As discussed above, the COX-2 pathway is implicated in colorectal cancer biology and it appears under the control of growth factor signal transduction in several experimental systems (DuBois *et al*, 1994; Di Popolo *et al*, 2000). Thus, it is a conceptually reasonable combination therapy that targets simultaneously COX-2 and growth factor receptor pathways (Figure 1). Recently, both a specific monoclonal antibody against Her-2/neu activity (Herceptin) and a selective EGFR-tyrosine kinase inhibitor (Iressa) came into clinical practice in cancer patients. The above molecules have been shown to synergise *in vitro* and in animal models the effects of conventional anticancer agents (Ciardiello *et al*, 2000). First, treatments based on combination therapy with NSAIDs and compounds that target other oncogenic pathways have been successfully tested in chemopreventive settings: Torrance *et al* (2000) showed that combination treatment of APCmin mice with sulindac and the ErbB tyrosine kinase inhibitor EKI-569 resulted in synergistic antitumour activity that leads to complete polyp prevention in half of all treated mice. Then, DuBois and collaborators, in the field of cancer therapy, clearly showed that the combination of celecoxib and herceptin had additive effects against the HCA rectal adenocarcinoma cell line *in vitro* and in xenograft, which leads to the almost complete inhibition of tumour growth (Mann *et al*, 2001).

Thus, coadministration of a selective COX-2 inhibitor with a tyrosine kinase inhibitor could enhance their single-agent anticancer activity and should be evaluated as a therapy *in vitro* and in colon cancer patients. At the moment, there are no trials designed to evaluate any of the above-mentioned combinations in colon cancer patients.

## CONCLUSIONS

Since the cure rate for colorectal cancer is low, it is mandatory to develop therapeutic strategies with less toxicity also for adjuvant therapy. Several lines of evidence suggest that COX-2 inhibitors not only counteract the development of malignant tumour at an early stage and cause premalignant tumours to regress, but also stimulate the death of established cancer cells. Therefore, COX-2 is becoming an attractive target for therapeutic strategies in colorectal cancer. The ability of NSAIDs and COX-2 inhibitors to synergise with conventional anticancer drugs and to induce apoptosis also independently from COX-2 expression *in vitro* further encourages their use in clinical practice. In addition, COX inhibitors are effective in reducing angiogenesis, supporting their use also in adjuvant settings. Recent evidences indicate that COX-2-selective inhibitors may also synergise with new inhibitors of the growth factor signal transduction pathways. Taken together, all this information provides a rationale for NSAIDs used alone or in combination with chemotherapeutic agents within clinical trials in colorectal cancer patients.
